# Characterization of SARS-CoV-2 genetic evolution in vaccinated and non-vaccinated patients from the Kenyan population

**DOI:** 10.21203/rs.3.rs-3457875/v1

**Published:** 2023-10-27

**Authors:** Samuel Oyola

**Affiliations:** International Livestock Research Institute

**Keywords:** Vaccination, unique mutations, single nucleotide polymorphism (SNP), Viral recombination, SARS-CoV-2, non-vaccinated

## Abstract

Vaccination is a key control measure of COVID-19 by preventing severe effects of disease outcomes, reducing hospitalization rates and death, and increasing herd immunity. However, vaccination can affect the evolution and adaptation of SARS-CoV-2, largely through vaccine-induced immune pressure. Here we investigated the recombination events and single nucleotide polymorphisms (SNPs) on SARS-CoV-2 genome in non-vaccinated and vaccinated patients in the Kenyan population. We identified recombination hotspots in the S, N, and ORF1a/b genes and showed the genetic evolution landscape of SARS-CoV-2 by comparing within-wave and inter-wave recombination events from the beginning of the pandemic (June 2020) to (October 2022) in Kenya. An in-depth analysis of (SNPs) on the S, ORf1a/b, and N genes identified previously unreported mutations. We detected a minority variant in non-vaccinated patients in Kenya, that contained immune escape mutation S255F of the spike gene and showing a differential recombination pattern within the non-vaccinated patients. Detailed analysis of recombination between waves suggested an association between increased population immunity and declining risk of emergence of variants of concern. Overall, this work identified unique mutations in SARS-CoV-2 which could have significant implications for virus evolution, virulence, and immune escape.

## Introduction

Previous studies have implicated both minimal and significant increase in the intrahost diversity of viruses with vaccination ^1–3^. Vaccination is a critical mitigation factor in controlling the COVID-19 pandemic and in Kenya it began with adults in March 2021 and later proceeded to teenagers in November 2021 ^4^. As of April 2023, 23 million vaccines have been dispensed to approximately 12 million adults and 2 million children (below 18 years). Of the 12 million adults, 10 million were fully vaccinated, whereas 2 million had received one dose of the vaccine ^4^. Nonetheless, by 2023 only 37.2% of adults and 10.1% of children were vaccinated, which is lower than other countries globally such as the USA, where 78.9% of adults and 77.4% of children are vaccinated ^4^.

A recent study evaluating the immunity of SARS-CoV-2 with vaccine uptake in Kenya highlighted issues with refusal, hesitancy, and inequity in society ^5^. It was reported that vaccine refusal was mainly due to concerns over safety, efficacy, and religious and cultural beliefs ^5^. Therefore, there is a gap in attaining herd immunity in Kenya and a deficiency in studies investigating the evolution and adaptation of the virus and the host, which could highlight broader perspectives on the overall effects of vaccination and population immunity.

SARS-CoV-2, the etiological agent of COVID-19, is an enveloped single-stranded RNA virus belonging to the genus betacoronavirus, also comprising of SARS-CoV and MERS-CoV ^6,7^. This virus contains 10 open reading frames (ORFs) and four major structural proteins, namely, Spike (S), Membrane (M), Envelope (E), and Nucleocapsid (N) ^6,7^. Based on previous works, the S gene, ORF1a/b, and the N gene single nucleotide polymorphisms (SNPs) significantly affect the virus’s infectivity, transmissibility, and overall fitness ^7–10^. For example, the spike protein of the omicron variant has approximately 26–35 mutations, which could affect the protein’s ability to bind to ACE-2 ^11,12^. Also, the ORF-1 has the largest number of missense mutations in the *nsp-3* gene, affecting viral replication ^11,12^

Evaluation of SNPs is a well-established approach to determine viral adaptation, however, identifying the RNA recombination events that may introduce deletions or insertions into the viral genome can be a step further in understanding viral evolution and its transmission dynamics during epidemics. SARS-CoV-2 is reported to contain both intrahost and intervariant recombination events and that the receptor-binding domain of SARS-CoV-2 is a product of recombination events between coronaviruses from pangolins ^13^. The current circulating Omicron variant, XBB and its descendant lineages XBB.1 and XBB.1.5, are thought to be a product of recombination between BA.2.10.1 and BA.2.75 variants ^14^. These intervariant recombinants are reported to have increased transmissibility and exhibit immune evasion, and ^14,15^. Numerous SARS-CoV-2 intrahost recombination events have been identified in clinical samples ^13,16–23^, therefore, an evaluation of both SNPs and recombination events can increase our understanding on the virus’ adaptation and evolution. This will further expand our knowledge of the biology behind the infectivity, clinical manifestation, and response to vaccines and therapeutics ^16,17^.

Here we investigate the evolution of SARS-CoV-2 in a cohort of vaccinated and non-vaccinated patients in Kenya. We identify intrahost recombination events in both groups and show similar trends in recombination patterns. We establish that the recombination ‘hotspots’ in both groups are found in the ORF1a/b, S, and N genes. We also show the recombination landscape of SARS-CoV-2 between and during transmission waves caused by different variants of concern in Kenya. We identify unique SNPs in vaccinated and non-vaccinated patients and, reveal a minority variant occurring in non-vaccinated patients, which could have immune escape properties. Overall, this work sheds light into the genetic evolution of SARS-CoV-2 in the Kenyan population and the effect of vaccination and introduction of new variants, through an in-depth analysis of both SNPs and recombination events.

## Results

### A cohort of vaccinated and non-vaccinated samples from COVID-19 patients in Kenya.

**Figure 1:**
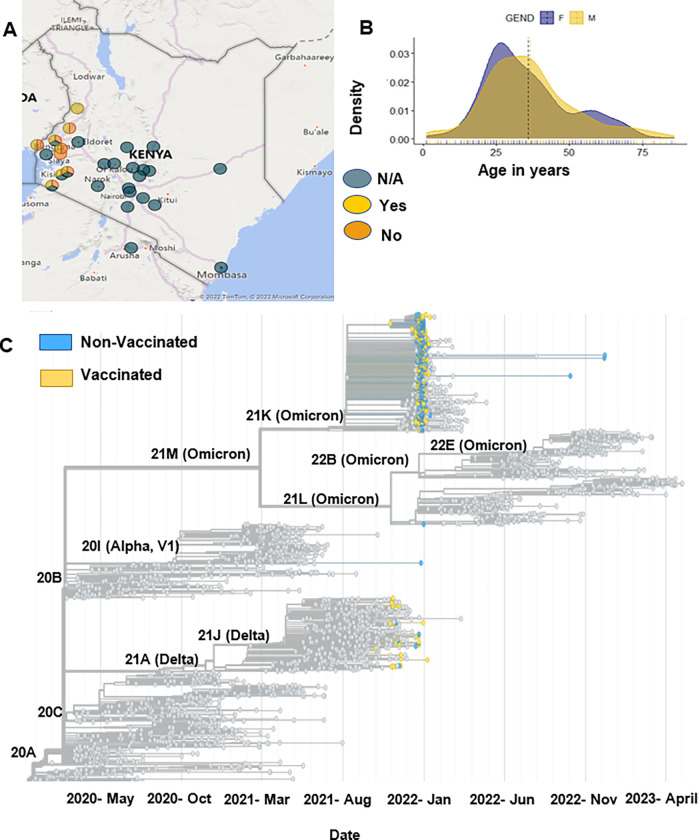
Demographic of vaccinated and non-vaccinated patients in Kenya. A. Shows the locations in Kenya of the vaccinated and non-vaccinated patients, dark blue circle shows samples with the vaccination status as not available (N/A), the yellow circle represents the vaccination status as yes, and the orange circle represents the vaccination status as no. B. Shows the age in years and gender of the cohort. C. Shows the phylogenetic analysis of non-vaccinated and vaccinated samples based on global trends.

A total of 601 SARS-CoV-2 samples collected from October 2021 to December 2022 were selected for analysis. This included 234 samples from non-vaccinated and 367 from vaccinated patients. All samples were reported to be SARS-CoV-2 positive based on RT-PCR testing and included information on the vaccination status as either yes or no. All samples were obtained from residents in the Kenyan counties of Bungoma, Busia, Homabay, Kakamega, Kisii, Migori, Nyamira, Trans Nzoia, Vihiga and West Pokot (Fig. 1A). The cohort contained 347 females and 254 males, with ages ranging between 20 and 50 (Fig. 1B).

We mapped this cohort to globally available sequences on UShER, where we show that the sequences were mainly from Delta and Omicron SARS-CoV-2 variants (Fig. 1C). This was expected based on the timing of sample collection. We identified the most frequently occurring SNPs in this cohort and noted that most genes are found on the S gene, ORF1a/b, and on the N gene (Supplementary Data 1). The most frequently occurring mutations in the S gene were D614G, H69_V70 deletion, T95I, G142_Y145 deletion, and T547. For the ORF1a/b gene, T3255I, L4715L, L3674_G3676 deletion, I3758V, and P3395H occurred the most, while in the N gene, P13L, E31_S33del, R203K, and G204R were the most occurring.

### Analysis of recombination events in vaccinated and non-vaccinated patients.

With an increase in genomic surveillance, SARS-CoV-2 recombination events of interest have been reported globally, making recombination a key factor in virus evolution ^16,20,22^. Recombination events were evaluated to determine SARS-CoV-2 genetic evolution in vaccinated and non-vaccinated patients. We used ViReMa, a viral recombination mapper that identifies intrahost recombination events including deletions, insertions, duplication, copy-back, snap-back, and viral-host chimeric events as described previously ^18,36,37^.

### An evaluation of SARS-CoV-2 transmission waves reveals differential recombination patterns

The possibility of inter-variant recombination was assessed. Following the pattern of SARS-CoV-2 transmission waves in Kenya ^41^, we grouped samples collected in to two categories (Table 1): Samples collected at the peak of transmission of a particular variant (lineage) and samples collected in the transition period between two variant waves of transition (interwave) (Fig. 3A) (Table 1). During the initial B.1 variant transmission wave, we detected an average of 688 deletions and 668 duplication events per patient, followed by a significant jump in the Beta transmission wave which had 2645 deletion, and 2676 duplication events. At the peak of Alpha variant transmission wave, we detected an average of 957 deletions, and 1050 duplication events. For the Omicron variant peak transmission wave, we observed 276 deletion and 247 duplication events (Fig. 3A) (Table 1).

The interwave 2 (Beta and Alpha) samples showed an average of 3877 deletion and 3904 duplication events per sample whereas interwave 3 (Alpha and Delta) had 4158 deletion and 4184 duplication events. Except for the peak transmission wave for Delta variant which showed the highest number of recombination events per sample (13629 deletion and 13700 duplication events), high number of recombination events were observed in samples collected during the interwave periods. This observation suggests the possibility of intervariant recombination events arising from mixed variant infection.

Next, we assessed recombination hotspots on the SARS-CoV-2 genome between and during the transmission waves and identified the location and frequency of recombination events. In the initial transmission waves, such as B.1 and interwave 1, we observe multiple locations and high frequency recombination events in different regions of the SARS-CoV-2 genome (Fig. 3B). Interestingly, however, the most recent variant (Omicron) had high frequency recombination hotspots mainly in the ORF1 a/b, S and N gene (Fig. 3B). We also noted an increase of recombination hotspots in most of the interwave periods of transmission compared to the transmission wave peaks. These observations suggest that over time, there was natural selection of recombination events in the ORF1 a/b, S and N gene and an increase of recombination event hotspots and frequency during mixed infection (interwave). This data reveals insights into the recombination activities within and between peak variants transmission waves.

### Analysis of SNPs between non-vaccinated and vaccinated patients reveals low-frequency unique non-synonymous mutations.

Recombination analysis identified genome positions of the four most common deletion events as 2883–2902 and 11286–11296 on the ORF1a/b, 21986–21996 on the S gene and 28362–28372 on the N gene. We analyzed the SNPs occurring in these recombination ‘hotspots’ to gain more insight on the genetic evolution in these regions. Of all the SNPs within the recombination hotspots, 66% in non-vaccinated and 69% in vaccinated patients were non-synonymous, whereas ≤ 34% were synonymous.

We also identified overlapping and unique SNPs, in the context of vaccinated and non-vaccinated. As shown on the Venn diagrams, 27% of all SNPs from vaccinated patients and 45% from non-vaccinated patients were unique to their vaccination status in the S gene (Fig. 4A). In the ORF1a/b gene, 32% of SNPs in vaccinated patients and 57% in non-vaccinated patients were unique to their vaccination status (Fig. 4B), and on the N gene, 42% of SNPs in vaccinated patients and 32% in non-vaccinated patients are unique to their vaccination status (Fig. 4C).

Further, we mapped all the unique non-synonymous SNPs on the S, ORF1a/b, and N genes to pinpoint their distribution on the functional domains of each gene product. As shown on the schematic representations of the gene products (Fig. 4B & 3C), mutations on the S gene were found to be distributed across the entire protein covering all the domains (Fig. 4A). Interestingly, however, on the ORF1a/b, we observed that all the unique mutations were concentrated within the N-terminal domain of the gene product, between *nsp1* and *nsp3* (Fig. 4B). This finding corroborates previous findings showing that *nsp-3* is the gene with the largest number of non-synonymous mutations on the ORF1a/b region of SARS-CoV-2, and mutations in this region have been shown to affect the virus papain like protease inhibitors, GRL-0617 and S43 binding capabilities ^42,43^. Like the S gene, the N gene unique mutations were distributed across the entire gene product (Fig. 4C).

### In-depth analysis of unique non-synonymous SNPs in the ORF1a/b, S, and N genes.

Focusing only on the non-synonymous unique SNPs in this cohort’s S, ORF1a/b, and N genes, as they are likely to cause changes in the protein function, we sought to determine whether the unique SNPs have been previously reported. Although low in frequency, our analysis of SNPs in the ORF1a/b identified new mutations that have not been reported elsewhere. In non-vaccinated patients, the identified mutations include G150C, P371S, H533Y, E743D, K1763R, S1856P, I3476V, L3919R, T4355I, L4460F, T4847I, N4969S, S5529F, L5624F, L6519R, M6580K, Q6843L, and A7014V. (Supplementary Fig. 3A). Of interest among these mutations was I3476V, which appeared in 11 non-vaccinated patients in Nyamira county in Kenya and was not found in any vaccinated cohort. In vaccinated patients, the new unreported mutations include V214A, I281T, A702T, H1141N, V1291F, K1202N, K2741E, A3615V, V3708L, T5355M, L6174S, and S6537F, (Supplementary Fig. 3A). Mutations with the highest frequency in this cohort were V214A (n = 6), A702T (n = 4), A3615V (n = 4), and V3708L (n = 4) (Fig. 4A).

Unique mutations were also identified in the spike protein. In non-vaccinated samples, we show that mutations T95L, I197T, Y200F, L229F, C432R, F429P, T732I, L858P, A958S, V1096A, I1198V, G1219C, and C1243G, are new and have not been reported before (Supplementary Fig. 3B). In vaccinated patients, we identified new unique mutations such V6I, A27V, T33I, G72E, T95V, R214S, A260D, F318I, R326N, P330S, S371I, K417Q, G431A, L552P, F565L, V622A, Q628K, T638A, V642G, I670V, M740I, P812Q, R847T, L959S, K1038E, F1062L, L1063P, Y1067H, K1086E, V1129A, C1243F, and G1246V (Supplementary Fig. 3B). Notably, SNP K417Q was found in position 417, that lies close to the interface of interaction between the Spike protein and ACE-2 receptors of the host. Several studies have shown this position to be mutated from a K to a T, however, in our samples it’s mutated from a K to a Q.

On the N gene, we found the following new mutations in non-vaccinated patients that have not been previously reported: A35V, L45S, D63Y, A173V, A182S, Q228H, M322V, S327L, T329A, K361E, and T362K (Supplementary Fig. 3C). Mutations D3G, T24N, Q28R, R36Q, R40H, Q70H, Y86H, I94T, A152V, A155S, R203fs, G204Q, S206fs, M210_A211_delins, A211S, G212fs, G214fs, Q289H, P344L, and K370N were unique to vaccinated patients (Supplementary Fig. 3C).

### Evaluation of a minority variant with linked co-mutations and recombination events

We sought to determine if the unique SNP mutations, based on vaccination status in the S, N and ORF1a/b genes occur in the same patient and if there was any correlation with recombination events. We evaluated the mutations based on the location of the patients, the number of patients in the cohort, and the frequency of recurring (Table 2). In the S gene of the non-vaccinated group, samples from Bungoma, Kakamega, Kisii, Migori, and Nyamira counties showed common mutations that are unique to non-vaccinated patients (Table 2). The most frequent mutation was S255F, found in 5 out of 8 patients in Nyamira county. We also identified mutation G1219C in 2 patients in Migori County. On the ORF1a/b genes, unique mutations were found in Bungoma, Kakamega, Migori, and Nyamira counties. The most frequently occurring mutation was I3476V, found in 10 out of 15 patients in Nyamira. Other mutations frequently occurring in Nyamira samples were N4969S (5/15), S5229F (5/15), and P1640L (4/15) (Table 1).

Mutations unique to vaccinated group of patients sampled from Bungoma, Kakamega, Migori, and Busia were also identified (Table 2). In the S gene, L212C (2/4) and R214S (2/4) were the most frequently occurring mutations in Kakamega. Whereas on the ORF1a/b gene, V2149A (6/19) was the most frequently occurring mutation event in Migori, followed by A3615V (4/19), V3708L (4/19), and V702T (4/19).

We next determined if these low-frequently unique SNPs in the S gene and the ORF1a/b co-occur in the same patients. Interestingly, we observed that in the non-vaccinated cohort, five samples in Nyamira had the same set of unique (only in non-vaccinated patients) mutations on S gene and the ORF1a/b (Fig. 5A). All five patients had mutations S255F on the S gene and I376V, N4969S, and S5339F on the ORF1a/b. S255F is an important mutation previously identified in the S gene, with immune escape properties ^1,4^, however, the I376V, N4969S, and S5339F are all unique to the non-vaccinated patients and have not been reported previously (Fig. 5A). This finding suggests a possible spread of a variant (minority variant) within a pocket of population and with an important immune evasion capability based on the presence of S255F.

To further characterize the evolution of this minority variant in this cohort, we analyzed the ViReMa recombination events in this group compared to other non-vaccinated patients (Fig. 5B). We noted that the top 5 recombination event positions for this cohort were 28247–28254, 76–26480, 75–27047, 4068–21432, 78–27769, and 18606–18985, which is different from 11286–11296, 2883–2902, 28362–28372, 21986–21996, 75–21562 found in other non-vaccinated patients (Fig. 5B). This observation portends that patients with this minority variant also had differential recombination events that could imply functional effects in virus’s adaptation, fitness, and infectivity.

## Discussion

The low uptake of COVID-19 vaccine in Kenya as with many other African countries could be attributed to, lack of trust in vaccine efficiency and safety, and religious beliefs ^5^. The low vaccination rate, and widespread infection-associated population immunity have limited studies seeking to understand the evolution and diversity of SARS-CoV-2 virus with respect to vaccine-induced immune pressure in the region. Here, we used next-generation sequencing to uncover the diversity and genetic evolution of SARS-CoV-2 through analysis of SNPs and recombination events with respect to vaccination status. Globally, recombination events have been reported in areas with high genomic surveillance, such as the UK, USA, and Denmark ^16,21–23,44^, and it is estimated that 5% of circulating US and UK SARS-CoV-2 viruses are recombinant ^16^. Hence genomic surveillance of SARS-CoV-2 by tracking both intervariant and intrahost recombination events has proven crucial in obtaining a better picture of the virus’ genetic evolution that may be driven by multiple variant infection, immune pressure, and vaccine efficacy; data that is critical in designing future control measures.

**Figure 2:**
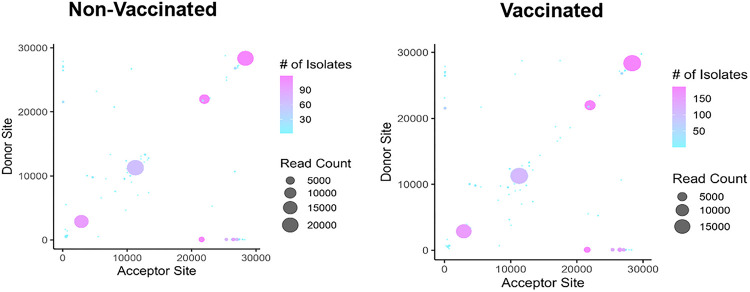
ViReMa identifies recombination events in vaccinated and non-vaccinated patients. The scatter plots show the mapping of recombination events of the 119 non-vaccinated and 187 vaccinated patients, where the donor site on the y axis is mapped to the acceptor site on the x-axis. The gradient in the legend of the scatter plot represents the number of patient samples containing at least a recombination event. The darker shaded circles in the scatter plot represent events that occur in multiple patient samples, while the circle size corresponds to the count of the reads of a recombination event.

**Figure 3:**
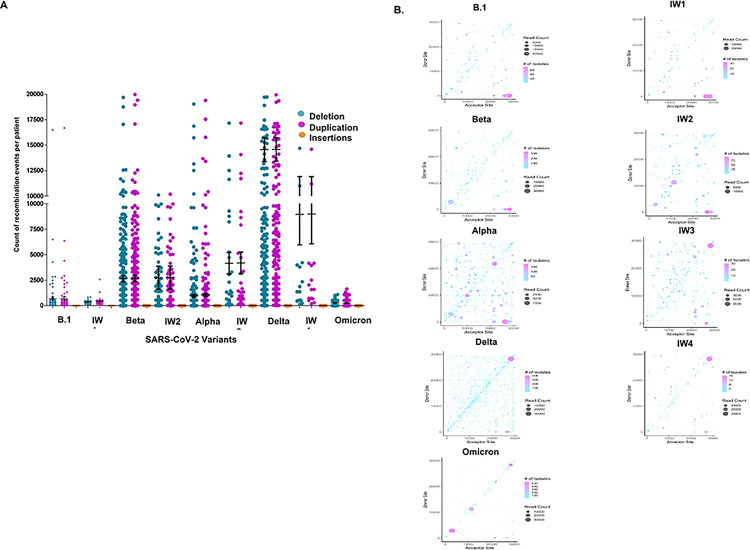
ViReMa identified recombination events between and during the peak of SARS-CoV-2 transmission waves. A. Shows the quantification of the recombination events per patients between and during SARS-CoV-2 variants. B. ViReMa scatter plots of SARS-CoV-2 recombination events and hotspots over B1, Beta, Alpha, Delta, and Omicron variants and the period between these variants interwave). The gradient in the legend of the scatter plot represents the number of patient samples containing a recombination event. The darker shaded circles in the scatter plot represent events that occur in multiple patient samples, while the circle size corresponds to the count of the reads of a recombination event.

**Figure 4:**
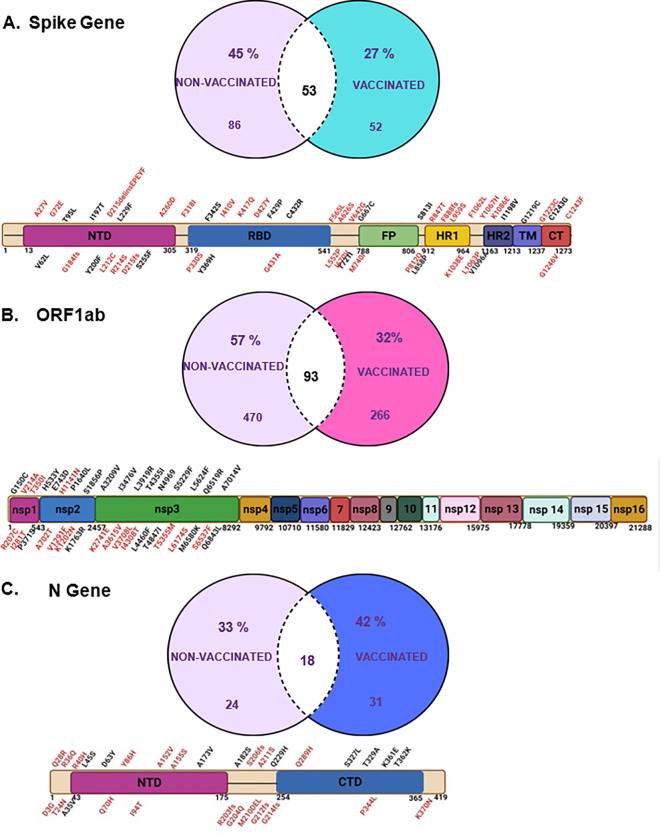
Analysis of unique non-synonymous SNPs on the S, ORF 1 a/b, and N genes in vaccinated and non-vaccinated patients. The Venn diagrams represent unique and overlapping SNPs between the samples from non-vaccinated and vaccinated patients. The schematics of the S, ORF1a/b, and N gene show the distribution of unique mutations across the domains of the SARS-CoV-2 gene products. Mutations written in black letters represent those found in non-vaccinated patients and those in red letters represent those in vaccinated patients.

**Figure 5:**
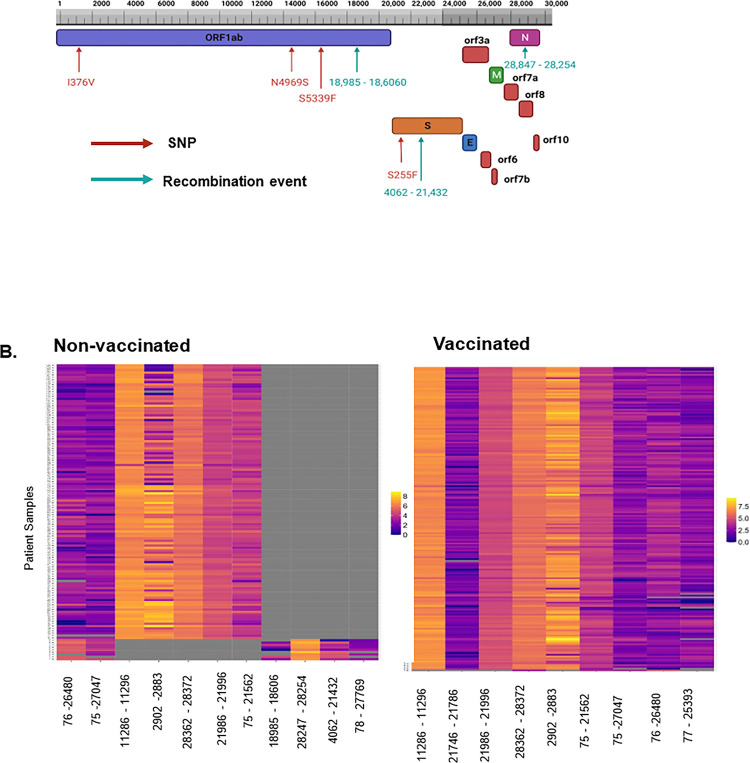
Analysis of the virus diversity of vaccinated and non-vaccinated patients reveals a minority variant. A. Schematic of the SARS-CoV-2 genome, highlighting co-occurring mutations on the S and ORF1a/b and top recombination events in patients in Nyamira county, Kenya. B. A heat map of log-transformed recombination events between the two groups (Vaccinated and non-vaccinated) showing differential recombination patterns in the patients with the minority variant.

With respect to vaccination status, we observed large number of recombination events in both vaccinated and non-vaccinated individuals (Fig. 2), which may correspond to the general high mutation rate of SARS-CoV-2 virus ^45^. We identify similar recombination ‘hotspots’ on the SARS-CoV-2 genome in vaccinated and non-vaccinated patient samples and show that the most common recombination events are found in the ORF1a/b, S, and N gene. These findings corroborate previous studies showing that recombination events occur disproportionately in the spike protein region, and that the ORF1a/b gene experiences the largest number of mutations and showing significant virus diversity in these regions ^16^.

The vaccine inequity early in the COVID-19 pandemic phase (early to late 2021) meant that vaccines became widely available in sub-Saharan Africa (SSA) at approximately the same time the Omicron variant occurred (around Oct-Nov 2021). As studies demonstrated, by early 2022, population immunity was > 70% across most urban and rural communities in SSA, making it difficult as shown in our studies, to discern the impact of vaccination in SARS-CoV-2 evolution [3]. Additionally, epidemiological data from these studies showed that both vaccinated and non-vaccinated patients had comparable immunity and thus exerting similar immunologic pressures on circulating SARS-CoV2 strains. While we observed comparable types and frequencies of recombination among vaccinated and non-vaccinated patients, the overall findings present a unique impact of immunologic pressure on the virus. Our data suggest that the delayed vaccination likely minimal onlyinduced strong antival immune pressure capable of driving genetic evolution. Instead, these findings reflect the impact of the combined effect of vaccination and widespread natural-infection-associated immunity in Africa.

We mapped the recombination landscape of SARS-CoV-2 between and within the peaks of variant transmission waves in Kenya. We showed significant differences in the average recombination events per patient and the quantity and frequency of recombination hotspots present during and between transmission waves. Interestingly, B.1 and Alpha variants produced the lowest recombination events at the peak of variant transmission wave, whereas Beta and Delta variants produced the highest recombination events at the peak of transmission wave. More recombination events were observed in samples within the peak of Beta variant transmission wave compared to Alpha. Similarly, samples collected within Delta variant transmission wave showed higher recombination events compared to Omicron. Given the transmission rate and virulence levels of these variants where Beta and Delta were generally viewed as having higher transmission rate and more virulence compared to Alpha and Omicron respectively ^46–48^, the correlating levels of recombination events observed could possibility be a functional pointer to the observed variant phenotypes. The peaking level of recombination in Delta variant before decreasing in the Omicron variant could also suggest that population immunity was associated with declining risk of emergence of variants of concerns.

Analysis of recombination events occurring in samples collected between two variant transmission waves (interwaves) corroborated the within wave observations. The highest interwave recombination events were observed between Beta to Alpha and Delta to Omicron transitions. B.1 to Beta and Alpha to Delta interwave recombination events were the lowest. Other than increased recombination events, these interwaves had multiple high frequency recombination hotspots compared to the transmission wave peaks. The observed increase of recombination events and hotspots could be correlated to multiple variant infections. A recent study evaluating spike protein diversity of omicron variants revealed shared mutations between omicron and other variants of concern and of interest ^15^. Conclusions from this previous work suggested that the confection of different SARS-CoV-2 variants leads to genome recombination which plays a key role in the ongoing genetic evolution of SARS-CoV-2.

We observed multiple high frequency hotspots in earlier variants such as B.1, compared to the most recent variant Omicron, which mostly adopted the use of ORF1a/b, S, and N gene. We hypothesize that SARS-CoV-2 evolution from B.1 to Omicron has led to the natural selection of the ORF1 a/b, S, and N gene as recombination hotspots. Several studies have highlighted the importance of these genes in the adaptability, transmissibility, and clinic outcomes of the Omicron variant infections ^15,46,48,49^. Overall, this recombination analysis shows that changes in the recombination landscape are increasingly affected by the circulating variant and epidemiological time point more than the vaccination status.

Globally, studies have identified types of SNPs occurring in variants of concern and variants of interest and how these mutations can affect the fitness of the virus and response to vaccines and anti-virals ^12,43,50,51^. SNP analysis gives insight into the regions and domains affected, and SARS-CoV-2 genomic positions and protein domains associated with virulence. For example, mutations such as S255F ^8,52^, confer reduced neutralization by monoclonal antibodies and have the potential for immune escape ^1,53,54^. In this study we identify unique SNPs, in the context of vaccination status Some mutations of interest include S255F, which occurred in a group of patients who had previously unreported SNPs in their ORF1a/b and showed a different pattern of recombination events compared to other non-vaccinated individuals. Also, we identified SNP K417T on position 417, one of the most frequently mutated positions and close to the spike-ACE-2 interaction interface ^8,9^. This mutation has often beenreported as a K to N, however, in our dataset it occued as a K to T mutation. In future works, it would be useful to further characterize the effects of these unique mutations with more functional experiments.

Of more interest is the detection of a minority variant in a small pocket of non-vaccinated population that seeks further studies and epidemiological follow-up. These patients from Nyamira county in Kenya appeared to have S255F mutation in the spike gene and new unreported mutations I376V, N4969S, and S5339F on ORF1a/b. Although S255F has been previously reported in the literature to cause immune evasion, ^4,51,59^ within an epidemiological cohort, its co-occurrence frequency with the other identified SNPs in this population is unique and may be a pointer to functional adaptational mechanisms of the virus. Additionally, we show within this same cohort, differential patterns of recombination events compared to the other non-vaccinated samples. These unique recombination patterns differentiate this cohort of samples from the rest of the population. This is a case study that further highlights the potential and importance of using SNPs and recombination analysis in unravelling viral genetic evolution and diversity.

Overall, the current study shows a broad picture of the differential virus genetic evolution and diversity between vaccinated and non-vaccinated patients and suggests increased recombination events and hotspots driven mostly by interaction between variants and little or no effect from the COVID-19 vaccines. Analysis of recombination events during peaks of transmission waves and the interwaves is a powerful approach to studying virus genetic evolution and its drivers. The work also demonstrates a methodology for studying genetic changes in a pathogen by a simultaneous analysis of both single nucleotide polymorphisms and recombination events.

## Materials and Methods

### Sample collection

We performed sample collection, processing, and analysis in accordance with the Ministry of Health-Kenya COVID-19 pandemic surveillance protocols and guidelines ^24^. The study samples were collected over the year 2020 to 2022 and comprised of nasopharyngeal and oral swabs. The samples were kept in viral transport media (VTM) tubes and transported under refrigerated conditions to the ILRI genomics laboratories in three tier packaging systems for processing. An aliquot of 300 microliters was used for RNA extraction, and the remaining was archived in the ILRI’s AZIZI Biorepository.

### SARS-CoV-2 RNA extraction

RNA extraction and purification was performed using the Tan Bead Nucleic RNA extraction kit (Opti Pure Viral Auto tube/plate) (Taiwan Advanced Nanotech Inc. Taoyuan City, Taiwan) following the manufacturer’s instructions. RT-qPCR was performed to identify SARS CoV-2 positive samples using Applied Biosystems Quant Studio 5 Real-Time PCR System (Thermos Fisher Scientific, USA).

### SARS-CoV-2 COVID-Seq Illumina library preparation and sequencing

RT-qPCR positive samples were then selected based on a CT < 35 and transitioned for library preparation. Purified RNA was used as template to prepare complementary DNA (cDNA) using random hexamers in a two-step reverse transcriptase process (Illumina COVID-Seq Ruo Kits, Illumina, Inc, USA) ^25–27^. This was followed by tilling/amplification of cDNA using the multiplex ARTIC primer-pools CPP1 and CPP2 version 3 followed by illumina library preparation protocol that uses enrichment bead-linked transposons (EBLT) for fragmentation, size selection, adaptor ligation and PCR enrichment (Illumina COVID-Seq Ruo Kits, Illumina, Inc, USA). The libraries were normalized and pooled to 4 nM before a further dilution to 1.5 pM for loading in NextSeq, or 12 pM for loading in MiSeq illumina sequencing platforms (Illumina, CA, USA) to sequence with the V2 paired-end chemistry ^25,26^

### Variant Calling and Consensus Genome construction.

The demultiplexed FASTQ files were merged for every sample and analyzed. Variant calling, and lineage assignment was performed using nf-core/viralrecon v2.5 - a Nextflow-based pipeline ^28,29^. Briefly, FASTQ files were quality filtered and adapter trimmed using FASTP v0.23.2 with a Phred Score cut-off of 20 ^30^. Bowtie2 v2.4.4 was used to map the reads to the reference genome (NC_045512.2) and iVar v1.3.1 to soft-mask primer sequences and identify the variants ^31,32^. SnpEff v5.06 and SnpSift v4.3 were used to annotate and filter relevant mutations identified ^33,34^. Re-construction of the consensus was done with bcftools v.1.15.1 ^35^.

### ViReMa recombination analysis

Virus-Recombination-Mapper (ViReMa; v0.25) was used to identify and quantify recombination events (insertions, deletion, and duplication) ^18,36,37^. We used paired-end next-generation sequence data to detect recombination junction events in the SARS-CoV-2 reference genome. ViReMa was run with default parameters plus the settings: -- *Seed 20* and --*MicroInDel_Length 5*. The recombination junction events, and their counts were reported in Browser Extensible Data (BED) files for each patient. For the vaccination data analysis, we retained samples with a genome coverage of > 99 and remained with 119 non-vaccinated and 187 vaccinated patient samples. Scatter plots showing ViReMa results were generated on the ViReMaShiny app and R ^38^. Scatter plots for both vaccination status and transmission waves included recombination events with a count number of > 10.

### Phylogenetic Analysis

Phylogenetic tree analysis using UShER and visualization using Nextstrain was done under the CZ Gen EPI tool ^39,40^. The CZ Gen EPI which is maintained by the Chan Zuckerberg Initiative and enabled by data from GenBank allows the generation and annotation of phylogenetic trees in Nextstrain. UShER provides a faster and more robust real-time analysis of the SARS-CoV-2 pandemic by ustilizing genomes from GISAID, GenBank, COG-UK, and CNCB ^40^. We uploaded our multifasta files onto CZ Gen EPI tool and build trees through the UShER option. Once the trees were completed, they were visualized on Nextstrain, and annotations on the samples included ^39^.

## Figures and Tables

**Table 1:** Shows the average deletion, duplication, and insertion events per patient in SARS-CoV-2 variants in Kenya.

Average events per patient	B.1 N = 97	Interwave 1 (IW1) N = 31	Beta N = 407	Interwave 2 (IW2) N = 142	Alpha N = 306	Interwave 3 (IW3) N = 38	Delta N = 442	Interwave 4 (IW4) N = 19	Omicron N = 574
**Deletion**	**688**	**364**	**2645**	**3877**	**957**	**4158**	**13629**	**8945**	276
**Duplication**	**668**	**441**	**2676**	**3904**	**1050**	**4184**	**13700**	**8674**	247
**Insertions**	**1**	**1**	**1**	**1**	**1**	**1**	**1**	**3**	1

**Table 2:** Patients with unique mutations in vaccinated and non-vaccinated patients in Kenya.

NON-VACCINATED				
SARS-CoV-2 Gene	Location	# of patients	Recurring mutations	Frequency
S GENE	BUNGOMA	23	NONE	0
	KAKAMEGA		NONE	0
	KISII	1	NONE	0
	MIGORI	2	G1219C	2/2
	NYAMIRA	8	S255F	5/8
ORF1ab	BUNGOMA	4	NONE	0
	KAKAMEGA	16	K1763R	2/16
			E743D	2
			H4533Y	2
			S1856P	3
			L3919R	3
	MIGORI	7	L5624F	2
			M6580K	2
	NYAMIRA	15	I3476V	10/15
			P1640L	4
			A3209V	2
			A7014V	2
			G150C	3
			N4969S	5
			S5229F	5
			T4355I	2
S GENE	BUNGOMA	12	NONE	0
	KAKAMEGA	4	L212C	2/4
			R214S	2/4
	MIGORI	14	R346N	2/14
	BUSIA	1	NONE	0
ORF1ab	BUNGOMA	3	I4308T	2/3
	KAKAMEGA	9	K2741E	2/9
			T350I	2/9
			K1202N	2/9
			L6174S	3/9
	MIGORI	19	A3615V	4/19
			V2149A	6/19
			V3708L	4/19
			V702T	4/19
	BUSIA	2	H1141N	2/2

## Data Availability

The Kenyan SARS-CoV genome sequence data used had been submitted to either global initiative on sharing avian influenza data (GISAID, https://www.gisaid.org/ accessed on 10 January 2022 or National Center for Biotechnology Information (NCBI, https://www.ncbi.nlm.nih.gov/).
